# Brief Report: Potent clinical and radiological response to larotrectinib in TRK fusion-driven high-grade glioma

**DOI:** 10.1038/s41416-018-0251-2

**Published:** 2018-09-17

**Authors:** David S. Ziegler, Marie Wong, Chelsea Mayoh, Amit Kumar, Maria Tsoli, Emily Mould, Vanessa Tyrrell, Dong-Anh Khuong-Quang, Mark Pinese, Velimir Gayevskiy, Richard J. Cohn, Loretta M. S. Lau, Mark Reynolds, Michael C. Cox, Andrew Gifford, Michael Rodriguez, Mark J. Cowley, Paul G. Ekert, Glenn M. Marshall, Michelle Haber

**Affiliations:** 1 Kids Cancer Centre, Sydney Children’s Hospital, Randwick, NSW Australia; 2 Children’s Cancer Institute, UNSW, Sydney, NSW Australia; 3 Kinghorn Centre for Clinical Genomics, Garvan Institute, Sydney, NSW Australia; 4 Peter MacCallum Cancer Centre, Melbourne, VIC Australia; 5 Murdoch Children’s Research Institute, Royal Children’s Hospital, The University of Melbourne, Melbourne, VIC Australia; 6 Clinical Development, Loxo Oncology, Inc., South San Francisco, San Francisco, CA USA; 7 Department of Anatomical Pathology, Prince of Wales Hospital, Randwick, NSW Australia; 8 St Vincent’s Clinical School, UNSW Sydney, Sydney, NSW Australia

**Keywords:** Targeted therapies, CNS cancer

## Abstract

Genes encoding TRK are oncogenic drivers in multiple tumour types including infantile fibrosarcoma, papillary thyroid cancer and high-grade gliomas (HGG). TRK fusions have a critical role in tumourigenesis in 40% of infant HGG. Here we report the first case of a TRK fusion-driven HGG treated with larotrectinib—the first selective pan-TRK inhibitor in clinical development. This 3-year-old girl had failed multiple therapies including chemotherapy and radiotherapy. Tumour profiling confirmed an *ETV6–NTRK3* fusion. Treatment with larotrectinib led to rapid clinical improvement with near total resolution of primary and metastatic lesions on MRI imaging. This is the first report of a TRK fusion glioma successfully treated with a TRK inhibitor.

## Introduction

High-grade gliomas (HGG) are highly aggressive brain tumours that affect both adults and children.^1^ The standard treatment is focal radiation therapy (RT), and the addition of temozolomide may prolong survival by a few months.^2^ HGG in infants has a better outcome with a survival rate of ~50%.^3^ Many respond to chemotherapy, however RT is with-held due to the impact of radiation therapy in infants.^4^ Treatment with RT may be used at recurrence, but with devastating sequelae. Progression following RT leads to a dismal outcome.^5^

Genes encoding neurotrophin receptor kinases (TRK) have recently been implicated as oncogenic drivers in multiple tumour types including infantile fibrosarcoma, papillary thyroid cancer and, rarely, adult HGG.^6,7^ Three members of the TRK proto-oncogene family have been described: TRKA, TRKB and TRKC, coded by the *NTRK1, NTRK2* and *NTRK3* genes, respectively.^8^ TRK fusions have been identified at varying frequency in paediatric gliomas, including in 3 out of 7 cases of infant HGG in one study, and have a critical role in tumourigenesis in mouse models.^9^

Larotrectinib is the first selective pan-TRK inhibitor in clinical development. Recent Phase 1 results showed that larotrectinib is well tolerated in children, with very high response rates in solid tumours that harbour TRK fusions.^10^ Conversely, solid tumours and HGG without TRK fusions were unresponsive to the inhibitor.^11^ Here we report the first case of a patient with a TRK fusion-driven HGG treated with a TRK inhibitor.

## Case report

The patient is a 3-year-old girl who was diagnosed with a brain tumour at 5 months of age. She presented initially with vomiting and seizures and an MRI showed a heterogeneous mass measuring 6 × 3 × 2 cm in the right lateral ventricle. Following gross total resection pathology showed predominance of large epithelioid and spindle-shaped cells with mild pleomorphism, mitotic index of 14 per 10 high power fields and a Ki67 proliferative index of 40%. The tumour showed patchy positivity for GFAP, strong nuclear staining for p53, and was negative for synaptophysin, chromogranin, NeuN, BRAF V600E, H3K27M and ATRX. She was diagnosed with a HGG and was treated with an infant brain tumour protocol with 13 cycles of chemotherapy.^5^

Four months after completing treatment, she had disease progression in the tumour bed with multiple nodules in the lateral and third ventricles. Further tumour debulking confirmed recurrent HGG. After 6 months, a new mass in the tumour bed was subtotally resected and she received focal radiotherapy of 54 Gy to the tumour bed. The resected tumour was profiled on a pilot personalised medicine study. Three months following completion of radiation therapy, she represented with difficulty walking, drowsiness, vomiting and irritability. MRI showed widespread progressive disease with increased enhancement at the resection site, and enlarging suprasellar and subependymal nodules in the lateral and third ventricles. Dexamethasone was continued at 1.5 mg daily. The parents were told that she was incurable, and she was referred to palliative care for symptom management.

## Results

**Fig. 1:**
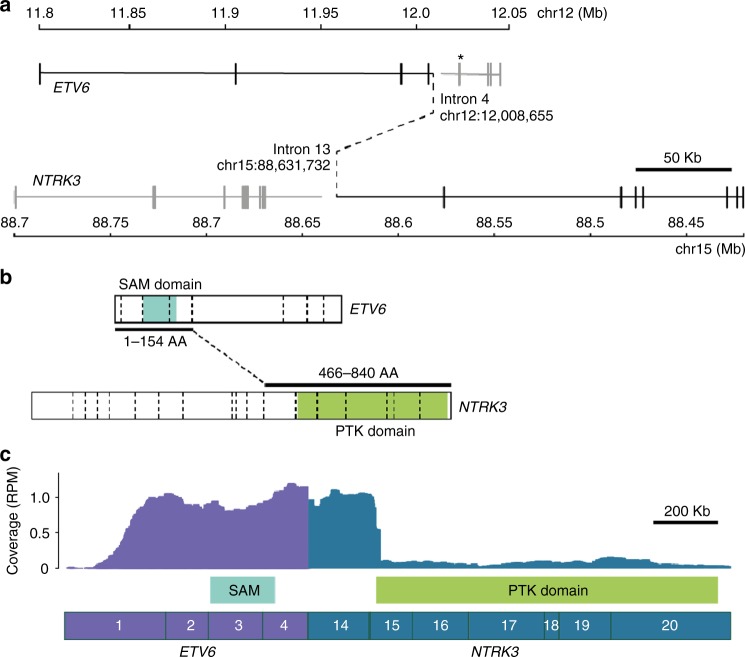
*ETV6–NTRK3* fusion. **a** Whole-genome sequencing revealed a t(12;15)(p13.2;q25.3) translocation, resulting in an in-frame *ETV6–NTRK3* fusion, denoted by black solid and dashed lines. The *ETV6*-breakpoint differs from the common *ETV6-RUNX1* translocation hotspot, which is indicated (*). **b** The first 154 *ETV6*, and last 374 *NTRK3* amino acids (AA) are fused, retaining *ETV6*′s sterile alpha motif (SAM) domain, as well as *NTRK3*′s protein tyrosine kinase (PTK) domain. Exon–exon boundaries are indicated with dashed vertical lines. **c** RNA-Seq confirmed the expression of the *ETV6–NTRK3* fusion, with 62 sequencing reads supporting the breakpoint junction. Exons are numbered. RPM: reads per million mapped reads. *ETV6* (NM_001987) and *NTRK3* (NM_001012338) isoforms used in all figures

Whole-genome sequencing of fresh-frozen tumour DNA (116× average depth) and matched germline DNA (43× average depth) revealed a t(12;15)(p13.2;q25.3) translocation, resulting in an *ETV6–NTRK3* fusion (Fig. 1a). The resulting fusion gene was in-frame, and retained the *ETV6* sterile alpha motif (SAM) domain, as well as the *NTRK3* protein tyrosine kinase (PTK) domain (Fig. 1b). RNA-Seq from fresh-frozen tumour RNA demonstrated that the *ETV6–NTRK3* fusion was robustly expressed (Fig. 1c). Immunohistochemistry staining with a pan-TRK antibody (monoclonal rabbit antibody EPR17341, Abcam) did not detect TRK expression.^12^ The tumour had a pathogenic *TP53* missense variant c.422G>A (p.Cys141Tyr), as well as somatic copy neutral loss of heterozygosity on chromosome 17, resulting in clonal biallelic loss of *TP53*, plus hemizygous loss of 9p and 18q, three copies of chrX, and biallelic focal deletion of *CDKN2A/B*. No germline mutations were identified.

Compassionate access to larotrectinib was obtained and commenced at a dose of 100 mg/m^2^ bd. After 4 weeks, she had no further lethargy, drowsiness, headaches or vomiting, was eating well, started talking clearly and had been weaned off dexamethasone. After 6 weeks, she was able to walk independently, was speaking in 2–3 word sentences, and had normal energy levels. By week 8, she was running, dancing, and continued to gain new words and language. No adverse events attributable to larotrectinib were observed.

**Fig. 2:**
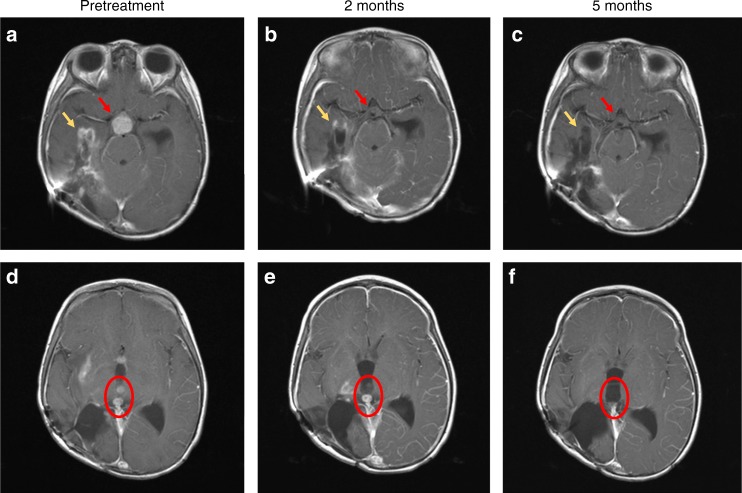
T1-weighted brain magnetic resonance imaging (MRI) with contrast images shown pre-treatment **a**, **d**, after 2 months of treatment **b**, **e** and after 5 months of treatment **c**, **f**. The contrast enhancing suprasellar mass (red arrows, **a**), had resolved after 2 months of treatment **b**, with sustained response at 5 months (**c**). Tumour bed enhancement (yellow arrows) improved at 2 months (**b**), and near complete at 5 months **c**. Examples of two contrast enhancing intraventricular lesions (red circle) pre-treatment **d**, improved after 2 months **e** and with complete resolution at 5 months **f**. The contrasting enhancing disease in the right thalamus are also visible in **e**, and had completely resolved after 5 months of treatment **f**

An MRI performed after 8 weeks of therapy showed resolution of the enhancing suprasellar mass, with improvement or resolution of all metastatic ventricular nodules and significantly less enhancement in the surgical bed. MRI at 5 months confirmed the response, with resolution of enhancement in the tumour bed and almost all metastatic lesions (Fig. 2). As of the time of this report, at 9 months from the start of larotrectinib, the patient continues on treatment with no adverse events.

## Discussion

To our knowledge, this is the first reported case of treatment with, and response to, a TRK inhibitor, in a patient with a TRK fusion-driven HGG. The marked response opens a new paradigm in the management of patients with this aggressive brain tumour. While 50% of infants with HGG respond to systemic chemotherapy, progression following chemotherapy and radiotherapy remains incurable, with dismal outcomes.^3,5^ The response to larotrectinib following failure of multiagent chemotherapy and radiotherapy suggests that TRK inhibitor therapy should be tested in the treatment of other TRK fusion-driven gliomas.

There are several TRK inhibitors currently in development, but no clinical results have till now been published in patients with TRK-driven brain tumours. Entrectinib is a selective tyrosine kinase inhibitor of the TRK kinases, C-Ros oncogene 1 and anaplastic lymphoma kinase. It has been shown to be active in a patient with a TRK-driven non-small cell lung cancer with cerebral metastases, suggesting the potential for this class of drugs to target primary intracranial tumours.^13^ In the recent report on larotrectinib efficacy in paediatric and adult clinical trials, 75% of patients with a TRK-driven tumour had an objective response to therapy.^14^ However, no patients were reported who had brain tumours harbouring TRK fusions, and the utility of this inhibitor for these HGG patients has, until now, been unknown. This report indicates that larotrectinib penetrates the BBB and may have potent activity in TRK-driven HGG.

It is unclear whether the activity seen in this case can be translated to adults with HGGs that harbour TRK fusions. TRK fusions in adult HGG are rare,^6^ however, given the paucity of effective treatment options for these patients, the results here suggest that biomarker-driven trials of TRK inhibitors are warranted in adult HGG patients.

How can TRK inhibitors be incorporated into the treatment of TRK fusion-driven HGG? Most important will be the detection of fusions by immunohistochemistry, FISH or sequenced-based tumour profiling (targeted sequencing, or whole genome and transcriptome sequencing). The latter techniques provide an unbiased approach, and may identify fusions in atypical tumour types, or novel rearrangements which may not be detected with standard tests. Notably, the pan-TRK antibody used here failed to recognise the ETV6–TRK3 fusion in this case, similar to a recent analysis.^15^ Further assessment of larotrectinib in clinical trials of HGG patients is needed to determine whether genomic profiling should be considered early in patient work-up. More evidence is also required to integrate TRK inhibitors into standard treatment, especially as some infants have durable responses to first-line chemotherapy. TRK inhibitors could be trialled in infants with relapsed or progressive disease, and as a strategy to avoid the damaging effects of high-dose radiation therapy, or combined with chemotherapy at diagnosis. The potential for integration of this molecularly driven, targeted therapy into the treatment for patients with HGG warrants further testing in clinical trials.

## **Data availability:**

All data generated or analysed during this study are included in this published article.
